# Moving Bragg grating solitons in a semilinear dual-core system with dispersive reflectivity

**DOI:** 10.1038/s41598-017-04179-6

**Published:** 2017-06-22

**Authors:** S. A. M. Saddam Chowdhury, Javid Atai

**Affiliations:** School of Electrical and Information Engineering, The University of Sydney, Sydney, NSW 2006 Australia

## Abstract

The existence, stability and collision dynamics of moving Bragg grating solitons in a semilinear dual-core system where one core has the Kerr nonlinearity and is equipped with a Bragg grating with dispersive reflectivity, and the other core is linear are investigated. It is found that moving soliton solutions exist as a continuous family of solutions in the upper and lower gaps of the system’s linear spectrum. The stability of the moving solitons are investigated by means of systematic numerical stability analysis, and the effect and interplay of various parameters on soliton stability are analyzed. We have also systematically investigated the characteristics of collisions of counter-propagating solitons. In-phase collisions can lead to a variety of outcomes such as passage of solitons through each other with increased, reduced or unchanged velocities, asymmetric separation of solitons, merger of solitons into a quiescent one, formation of three solitons (one quiescent and two moving ones) and destruction of both solitons. The outcome regions of in-phase collisions are identified in the plane of dispersive reflectivity versus frequency. The effects of coupling coefficient, relative group velocity in the linear core, soliton velocity and dispersive reflectivity and the initial phase difference on the outcomes of collisions are studied.

## Introduction

Fiber Bragg gratings (FBGs) are widely used in many applications in optics and optical communications such as optical filters, format conversion, sensors and dispersion compensation^1–6^. FBGs are also promising candidates for various nonlinear applications such as optical switching and pulse compression^7–11^. One of the main characteristics of FBGs are their strong dispersion due to cross-coupling between forward- and backward-propagating waves, which can be up to six orders of magnitude greater than the underlying chromatic dispersion of silica^12, 13^. At high intensity, the nonlinear effect of the fiber can balance out the grating induced dispersion, resulting in the formation of Bragg grating (BG) solitons (strictly speaking, these pulses are robust solitary waves). BG solitons in Kerr nonlinear media have been investigated extensively both theoretically^14–16^ and experimentally^17–20^ over the past few decades. It has been found that BG solitons form a two-parameter family of solutions that exist through out the bandgap of the linear spectrum of the grating^13–16^. Also, analytical and numerical analyses have shown that nearly half of the soliton family is stable against oscillatory perturbations^21–23^.

An important property of BG solitons is that they can propagate at any velocity in the range zero and the speed of light in the medium. This intriguing feature has led to a great deal of experimental efforts to be devoted to the generation of slow BG solitons owing to their potential use in various slow light applications such as in optical delay lines, optical buffers and logic gates. To date, BG solitons with velocities as low as 23% of the speed of light in the medium have been experimentally observed^24^. Similar solitons have been investigated in more sophisticated photonic structures such as waveguide arrays^25^, photonic crystals^26, 27^ and dual core systems^28–34^. They have also been explored in diverse nonlinear media such as quadratic nonlinearity^35, 36^, sign-changing Kerr nonlinearity^37^ and cubic-quintic nonlinearity^38, 39^. It is worth noting that transitional radiation in an optical lattice may prevent the formation of moving solitons^40^. There are systems where solitons with discrete set of propagation velocities can exist due to complete suppression of transitional radiation^41, 42^. In systems where the dynamics can be approximated by coupled counterpropagating waves (e.g. in a Bragg grating), the transitional radiation can be exponentially small and therefore moving solitons can exist.

In more complex Bragg grating structures, such as nonuniform gratings, Bragg super-structures^43, 44^ and gratings written on photonic wires^45^, reflection spectra may feature broad and inhomogeneous bandgaps. In such cases, the effect of nonuniformity needs to be considered and therefore the standard model of BG solitons must be modified. One possible approach, proposed in ref. 46, is to incorporate the contribution of spatial dispersion stemming from the random variations of Bragg reflectivity (aka dispersive reflectivity) into the standard model. The dispersive reflectivity model is a phenomenological generalization of the standard model, which may be applicable to weakly disordered gratings (i.e., gratings with random nonuniformities). It has been shown that dispersive reflectivity on the stability has a stabilizing effect on BG solitons^46, 47^.

Optical fiber couplers, particularly the ones with mismatched or nonidentical cores (e.g., semilinear couplers) have been the subject of much interest over the past three decades owing to their potential applications in switching and signal processing^48–55^. Dual core systems equipped with Bragg gratings (e.g., grating assisted couplers) are strong candidates for optical add/drop elements in WDM systems^56–60^. In the context of BG solitons, it has been shown that semilinear dual core system with an embedded uniform or nonuniform BG only in the nonlinear core can support both quiescent (zero velocity) and moving BG solitons^32, 34^. In another study, it has been numerically demonstrated that a grating-assisted semilinear coupler can support very slow BG solitons^61^. Such structures can be produced with current technology by writing a uniform or nonuniform grating in one of the two cores^56–58^. BG solitons in dual core systems can be a potential candidate for various novel optical devices (e.g., all-optical soliton diode reported in ref. 62).

Interactions and collision of solitons have been investigated in different systems due to the fact that outcomes of collisions provide a better insight into the intricacies of the system^63–65^. In the case of BG solitons, it has been theoretically shown that solitons can be trapped inside a Bragg grating through collisions with an appropriately designed localized defect^66–68^. Other studies have suggested a mechanism to create zero velocity or very slow moving light pulses through collisions of counter-propagating solitons in a Bragg grating^47, 69, 70^. It has also been shown that collisions and interactions of solitons in Bragg gratings can be exploited to perform optical logic gates^71^.

In this paper, we investigate the existence, stability and collision dynamics of moving BG solitons in the model introduced in ref. 34. In the Section of Results and Discussion, the model and its linear spectrum in the moving reference frame are presented and discussed. We then analyze the existence and stability of the moving solitons in the model. Next, the outcomes of collisions are analyzed and discussed. Finally, a summary of the results and conclusions are provided.

## Results and Discussion

### The model and its linear spectrum

**Figure 1:**
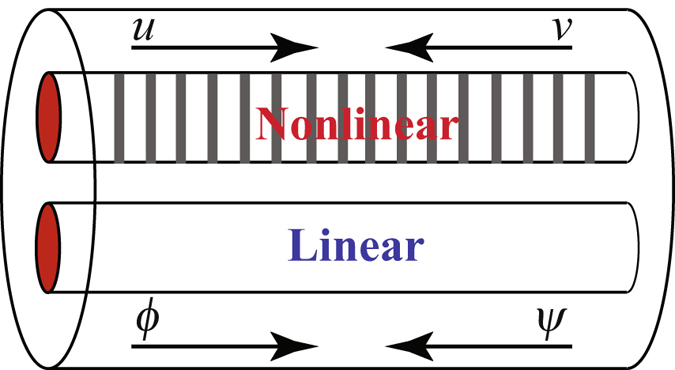
Schematic diagram of a semilinear dual-core system where a nonlinear core with a Bragg grating is coupled with a linear one.

In ref. 34 a model for a semilinear dual core system was put forward which comprised a nonlinear (Kerr) core equipped with a Bragg grating with dispersive reflectivity and a uniform linear core. The system is shown schematically in Fig. 1.

Starting from Maxwell’s equation and following the procedure for the derivations of coupled mode equations (see for example Chapter 3 in ref. 72) and dispersive reflectivity^46^, one can arrive at the following system of partial differential equations:

1 $$\begin{array}{rcl}\,i{u}_{t}+i{u}_{x}+[{|v|}^{2}+\frac{1}{2}{|u|}^{2}]u+v+\kappa \varphi +m{v}_{xx} & = & 0\\ i{v}_{t}-i{v}_{x}+[{|u|}^{2}+\frac{1}{2}{|v|}^{2}]v+u+\kappa \psi +m{u}_{xx} & = & 0\\ \quad \quad \quad \quad \quad \quad \quad \quad \quad \quad \,\,\,i{\varphi }_{t}+ic{\varphi }_{x}+\kappa u & = & 0\\ \quad \quad \quad \quad \quad \quad \quad \quad \quad \quad \,\,i{\psi }_{t}-ic{\psi }_{x}+\kappa v & = & 0\end{array}$$

where *u* and *v* denote the forward- and backward-propagating waves, respectively, in the nonlinear core, and *ϕ* and *ψ* are their counterparts in the linear core. Also, *κ* is the coefficient of linear coupling between the two cores, *m* represents the strength of dispersive reflectivity and *c* is the relative group velocity in the linear core with respect to the group velocity in the nonlinear core. As the values *m* > 0.5 may not be physically meaningful^47^, we confine our analysis to the range 0 ≤ *m* ≤ 0.5 without the loss of generality.

As far as the realization of the model is concerned, two main parameters are the coupling length, *L*_*c*_, and Bragg reflection length, *L*_*B*_ which should be of the same order of magnitude. *L*_*B*_ is typically ~1 mm. Couplers can also readily be manufactured such that *L*_*c*_ ~ 1 mm. Δ*t* = 1 and Δ*x* = 1 correspond to 10 ps and 1 mm in physical units. Since the nonlinear coefficient of silica ~2 (kmW)^−1^, the peak power needed for the formation of solitons is estimated to be ~1 MW. This estimate is actually an upper bound. Experimental studies have shown that considerably less power is needed for the formation of solitons^17–20^. The above parameter estimates yield a length of ~10 cm for the dual-core fiber.

In order to find soliton solutions with nonzero velocities, Eq. (1) are first transformed into the moving coordinates using {*X*, *T*} = {*x* − *δt*, *t*}, where *δ* is the soliton velocity and is normalized such that *δ* = 1 corresponds to the speed of light in the medium:

2 $$\begin{array}{rcl}\,i{u}_{T}+i\mathrm{(1}-\delta ){u}_{X}+({|v|}^{2}+\frac{1}{2}{|u|}^{2})u+v+\kappa \varphi +m{v}_{XX} & = & 0,\\ i{v}_{T}-i\mathrm{(1}+\delta ){v}_{X}+({|u|}^{2}+\frac{1}{2}{|v|}^{2})v+u+\kappa \psi +m{u}_{XX} & = & 0,\\ \quad \quad \quad \quad \quad \quad \quad \quad \quad \quad \quad \,\,i{\varphi }_{T}+i(c-\delta ){\varphi }_{X}+\kappa u & = & 0,\\ \quad \quad \quad \quad \quad \quad \quad \quad \quad \quad \,\,\,\,\,i{\psi }_{T}-i(c+\delta ){\psi }_{X}+\kappa v & = & 0.\end{array}$$

To characterize the spectral gap within which moving solitons may exist, it is necessary to analyze the system’s linear spectrum. Substituting *u*, *v*, *ϕ*, $\psi \sim {e}^{ikX-i{\rm{\Omega }}T}$ into the linearized form of Eq. (1), we arrive at the following dispersion relation for Ω(*k*):

3 $$\begin{array}{l}{{\rm{\Omega }}}^{4}+4k\delta {{\rm{\Omega }}}^{3}-[1+2{\kappa }^{2}+(1+{c}^{2}-2m-6{\delta }^{2})\,{k}^{2}+{m}^{2}{k}^{4}]\,{{\rm{\Omega }}}^{2}\\ \quad -2k\delta [1+2{\kappa }^{2}+(1+{c}^{2}-2m-2\delta )\,{k}^{2}+{m}^{2}{k}^{4}]\,{\rm{\Omega }}\\ \quad +{\kappa }^{4}+[{c}^{2}-2c{\kappa }^{2}-{\delta }^{2}(1+2{\kappa }^{2})]\,{k}^{2}\\ \quad +[{c}^{2}(1-2m)-(1+{c}^{2}-2m)\,{\delta }^{2}+{\delta }^{4}]\,{k}^{4}\\ \quad +({c}^{2}-{\delta }^{2})\,{m}^{2}{k}^{6}=0,\end{array}$$

where Ω is the Doppler-shifted frequency-detuning in the moving frame and is related to *ω* in the laboratory (stationary) reference frame by

4 $${\rm{\Omega }}(k)=\omega (k)-\delta k.$$

**Figure 2:**
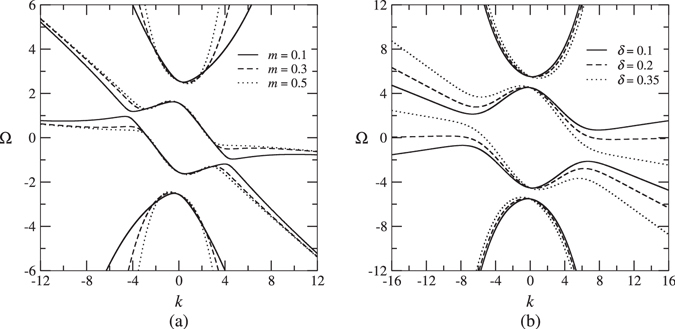
Dispersion diagrams for various values of *m* and *δ*. (**a**) *κ* = 2, *δ* = 0.25, and *c* = 0.2; (**b**) *κ* = 5, *m* = 0.3 and *c* = 0.2.

Note that for *δ* = 0, dispersion relation (3) reduces to the one given by Eq. (2) in ref. 34. Similar to the quiescent case (the model of ref. 34), when *c* = 0, there exist two disjoint bandgaps that reside in the upper and lower halves of the spectrum. Also, for $c\ne 0$, a central bandgap is formed. However, unlike the quiescent case, these bandgaps are not genuine ones because they overlap with one branch of continuous spectrum. Examples of dispersion diagrams are shown in Fig. 2.

In the cases where the bandgap is open, the edges of the bandgap Ω_*m*_ are determined by solving $d{\rm{\Omega }}(k)/dk=0$. However, the equation $d{\rm{\Omega }}(k)/dk=0$ does not yield an expression that can be solved analytically. Therefore, Ω_*m*_ must be determined numerically. It should be noted that, unlike the quiescent case, the edges of the upper and lower gaps never occur at *k* = 0 when $\delta \ne 0$. Also, Our analysis shows that the widths of the upper and lower gap reduce with increasing *δ* for fixed values of *m* and *c*. Similarly, increasing *m* for fixed *δ* and *c* results in the reduction of the width of the upper and lower gaps. A noteworthy finding is that there exists a critical velocity *δ*_*cr*_ at which the upper/lower gap closes. Based on the numerical calculations, the following empirical expression for the critical velocity has been deduced:

5 $${\delta }_{cr}=\frac{c+1}{2}.$$

**Figure 3:**
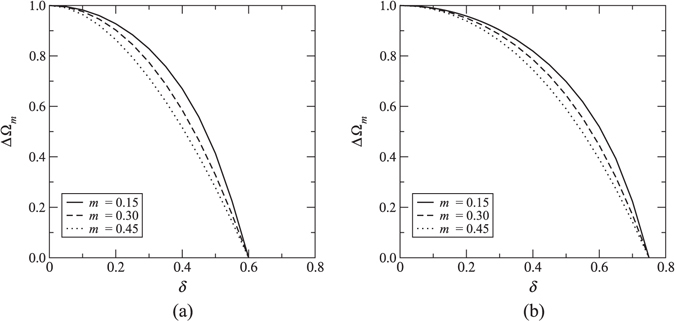
Width of the upper/lower gap ΔΩ_*m*_ as a function of velocity *δ* for different values of *m*. (**a**) *κ* = 10, *c* = 0.2; (**b**) *κ* = 5, *c* = 0.5.

Figure 3 shows how the width of the upper/lower gap ΔΩ_*m*_ varies with increasing *δ* for given values of *c* and *m*. It is worth noting that, consistent with the relation (5), ΔΩ_*m*_ becomes zero exactly at *δ* = 0.6 (Fig. 3(a)) and *δ* = 0.75 (Fig. 3(b)) for *c* = 0.2 and *c* = 0.5, respectively. Additionally, it is evident from this figure that ΔΩ_*m*_ is independent of *m* for *δ* = 0 and *δ* = *δ*_*cr*_.

### Soliton solutions and their stability

Soliton solutions of the system of Eq. (2) are sought as

6 $$\begin{array}{rcl}\,\{u(X,T),v(X,T)\} & = & \{U(X),V(X)\}{e}^{-i{\rm{\Omega }}T},\\ \{\varphi (X,T),\psi (X,T)\} & = & \{{\rm{\Phi }}(X),{\rm{\Psi }}(X)\}{e}^{-i{\rm{\Omega }}T}.\end{array}$$

Substitution of the ansatz (6) into (2) leads to the following system of ordinary differential equations:

7 $$\begin{array}{rcl}{\rm{\Omega }}U+i\mathrm{(1}-\delta ){U}_{X}+[{|V|}^{2}+\frac{1}{2}{|U|}^{2}]\,U+V+\kappa {\rm{\Phi }}+m{V}_{XX} & = & 0,\\ {\rm{\Omega }}V-i\mathrm{(1}+\delta ){V}_{X}+[{|U|}^{2}+\frac{1}{2}{|V|}^{2}]\,V+U+\kappa {\rm{\Psi }}+m{U}_{XX} & = & \mathrm{0,}\\ \quad \quad \quad \quad \quad \quad \quad \quad \quad \quad \quad \quad {\rm{\Omega }}{\rm{\Phi }}+i(c-\delta ){{\rm{\Phi }}}_{X}+\kappa U & = & \mathrm{0,}\\ \quad \quad \quad \quad \quad \quad \quad \quad \quad \quad \quad \quad {\rm{\Omega }}{\rm{\Psi }}-i(c+\delta ){{\rm{\Psi }}}_{X}+\kappa V & = & 0.\end{array}$$

To obtain the moving soliton solutions, Eq. (7) are solved numerically using a relaxation algorithm. It is found that, similar to their quiescent counterparts (cf. ref. 34), moving solitons exist only in the upper and lower gaps. Also, up to the available numerical accuracy, the soliton solutions form a continuous family of solutions. Another interesting feature of the moving soliton solutions is that sidelobes may appear in solitons’ profile in the presence of dispersive reflectivity. The formation of sidelobes, however, is dependent on the parameters *κ*, *c*, *m* and *δ*.

To gain a better understanding as to how various parameters affect the stability of solitons in Eq. (1), we first summarize stability characteristics of Bragg gratings solitons in the single-core case. In the case of *uniform* single-core Bragg grating, it has been shown that the stability of the family of moving solitons reported in ref. 16 is almost independent of the velocity of solitons^21, 22^. The presence of dispersive reflectivity in the single-core case (i.e. the model of ref. 46) results in stabilization of solitons which, in turn, leads to the expansion of the stability region within the bandgap. However, in this case, the stability border becomes dependent on the velocity of solitons. In the model of Eq. (1), the coupling between the cores and the group velocity mismatch strongly affect the stability of solitons. In particular, the stability analysis shows that the stabilization of solitons due to dispersive reflectivity depends on the strength of the coupling coefficient and can be counteracted by soliton velocity and group velocity mismatch parameter (see below).

**Figure 4:**
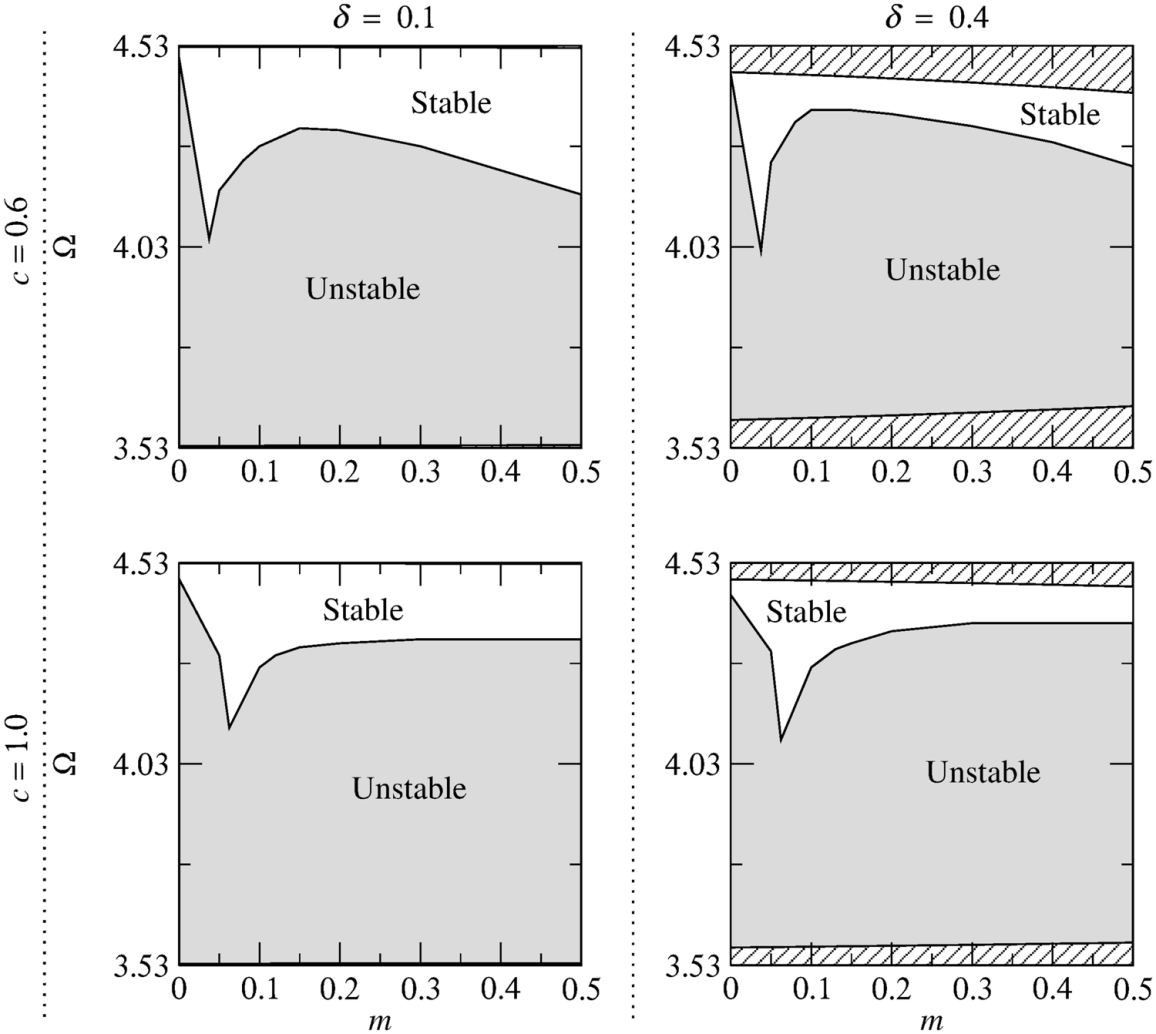
Stability regions corresponding to the upper gap in the (*m*, Ω) plane for different values of soliton velocity (increasing *δ* from left to right) and relative group velocity (increasing *c* from top to bottom) for *κ* = 4. The areas which lie outside the gap and consequently do not contain soliton solutions are shown by the diagonal lines.

**Figure 5:**
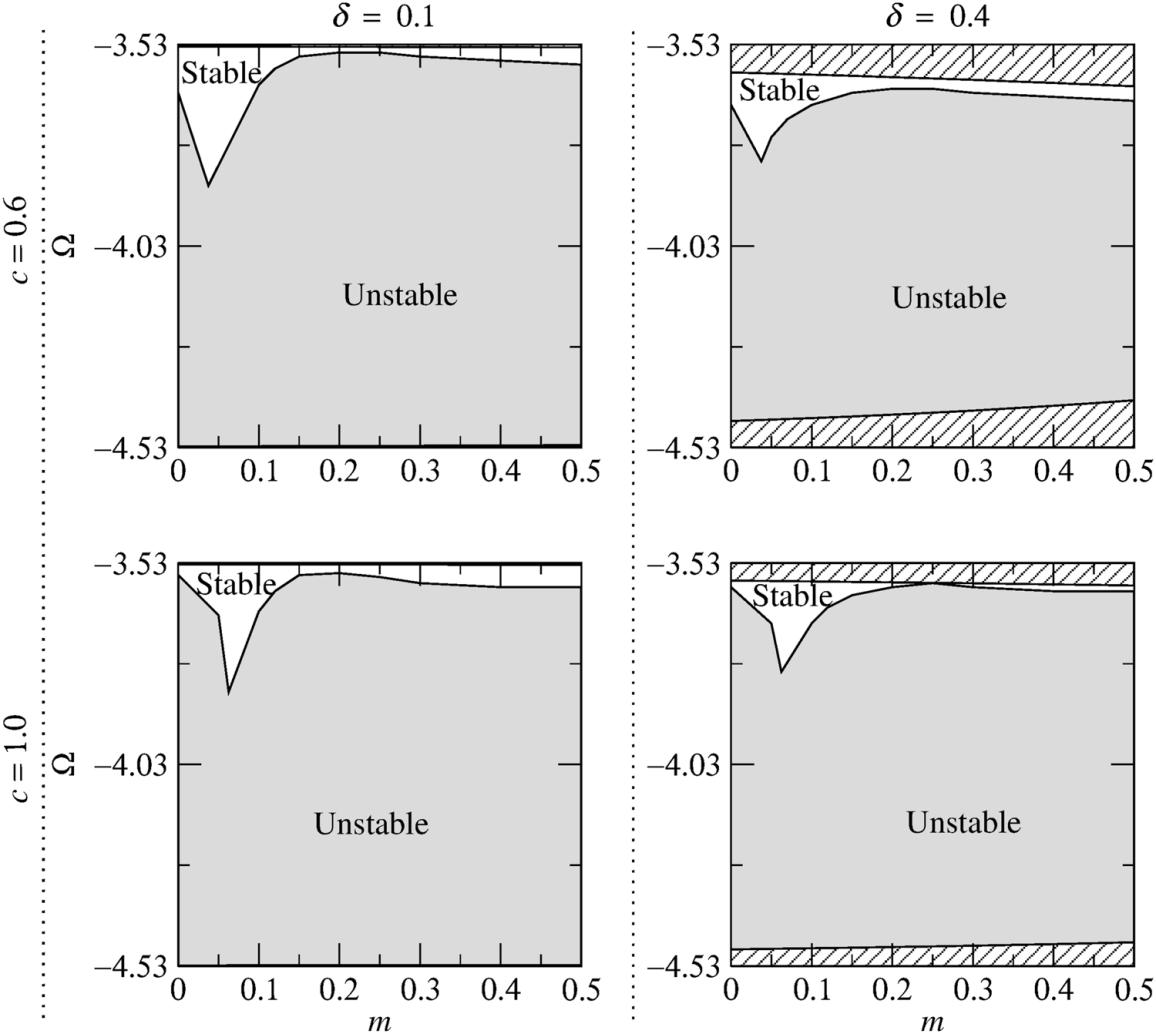
Stability regions corresponding to the lower gap in the (*m*, Ω) plane for different values of soliton velocity (increasing *δ* from left to right) and relative group velocity (increasing *c* from top to bottom) for *κ* = 4. The areas which lie outside the gap and consequently do not contain soliton solutions are shown by the diagonal lines.

To determine the stability of solitons in the model of Eq. (1), we have conducted a systematic numerical stability analysis by simulating their evolution using the symmetrized split-step Fourier algorithm^73^ for different values of *κ*, *c*, *m* and *δ*. To seed any inherent instability, the moving soliton solutions are initially perturbed asymmetrically. The results of the stability analysis are summarized in the (*m*, Ω) plane for *κ* = 4 in Figs 4 and 5 for the upper and lower gaps, respectively. The results for other values of *κ* (e.g., *κ* = 2, 5 and 10) are not shown separately but are included in Figs 9, 10 and 11. A general trend shown in these figures is that the stabilization effect due to dispersive reflectivity is dependent on *κ* and is more pronounced for large *κ* (e.g., *κ* = 10). However, an increase in *c* significantly affects the soliton stability for a given *κ* and *δ*, particularly in the lower gap (see, for example, Fig. 10). Furthermore, for a given *c* and *κ*, increasing *δ* results in the gradual reduction of the area of the stable region. An interesting feature of Figs 4 and 5 is that a cusp in the stability border is formed when *c* = *mκ* ^2^ regardless of the soliton velocity. Another notable finding is that, for a given *c* and *δ*, the stable region in the lower gap expands as *κ* becomes larger (see Fig. 11).

**Figure 6:**
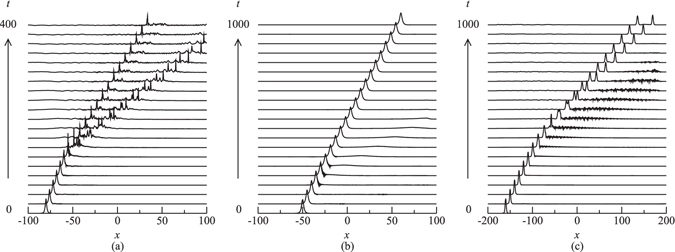
Examples of the evolution of the unstable solitons. (**a**) Ω = 9.86, *κ* = 10, *m* = 0, *c* = 0, and *δ* = 0.2; (**b**)  Ω = −9.89, *κ* = 10, *m* = 0, *c* = 0.2, and *δ* = 0.1; and (**c**)  Ω = 9.69, *κ* = 10, *m* = 0.3, *c* = 0.2, and *δ* = 0.2.

The unstable solitons do also exhibit interesting dynamics. The evolution of highly unstable solitons (i.e. solitons far from stability border) leads to a significant amount of radiation and subsequent destabilization and destruction of the soliton (Fig. 6(a)). The unstable solitons may shed some energy in the form of radiation and evolve to another stable moving soliton (Fig. 6(b)). There also cases where instability development results in the spontaneous splitting of the soliton into two stable moving ones (Fig. 6(c)).

### Collisions of solitons

To investigate the characteristics of soliton collisions, systematic simulations were performed by propagating stable counter-propagating moving solitons in the (*m*, Ω) plane for several values of *κ*, *δ* and *c* using the following conditions as initial input:

8 $$\begin{array}{rcl}u(X,\mathrm{0)} & = & {u}_{\delta }(X-\frac{{\rm{\Delta }}X}{2},0)+{u}_{-\delta }(X+\frac{{\rm{\Delta }}X}{2},0)\,{e}^{i{\rm{\Delta }}\theta },\\ v(X,\mathrm{0)} & = & {v}_{\delta }(X-\frac{{\rm{\Delta }}X}{2},0)+{v}_{-\delta }(X+\frac{{\rm{\Delta }}X}{2},0)\,{e}^{i{\rm{\Delta }}\theta },\\ \varphi (X,\mathrm{0)} & = & {\varphi }_{\delta }(X-\frac{{\rm{\Delta }}X}{2},0)+{\varphi }_{-\delta }(X+\frac{{\rm{\Delta }}X}{2},0)\,{e}^{i{\rm{\Delta }}\theta },\\ \psi (X,\mathrm{0)} & = & {\psi }_{\delta }(X-\frac{{\rm{\Delta }}X}{2},0)+{\psi }_{-\delta }(X+\frac{{\rm{\Delta }}X}{2},0)\,{e}^{i{\rm{\Delta }}\theta },\end{array}$$

where *u*, *v*, *ϕ* and *ψ* are the components of the moving soliton solutions and the subscript ±*δ* denotes the velocity at which the counter-propagating solitons are traveling. Also, Δ*X* is the initial separation and Δ*θ* is the initial phase difference between the solitons.

The results of simulations have shown that the soliton-soliton collisions give rise to some generic outcomes that are in common with those in uniform single-core Bragg gratings. More specifically, the collisions may result in the generation of a single quiescent soliton, two symmetrically separating solitons whose velocities are smaller, larger or the same as the original ones. Also, similar to the case of a single-core Bragg grating with dispersive reflectivity^47^, the collisions may also lead to the formation of a quiescent solitons and two moving ones (i.e. 2 → 3 transformation). A major difference between collisions in the model of Eq. (1) and those in ref. 47 is that in the case of single-core Bragg grating with dispersive reflectivity, the 2 → 3 transformation occurs for strong dispersive reflectivity whereas in the dual-core case considered in this paper the 2 → 3 transformation can occur for both strong and moderate dispersive reflectivity. Another noteworthy feature of the collisions is that their outcomes in the upper and lower bandgaps are not the same. These new features occur as a result of the coupling between the cores, group velocity mismatch and the initial velocity of solitons. Below, we analyze how the interplay of these parameters affects the dynamics of soliton-soliton collisions and their outcomes.

**Figure 7:**
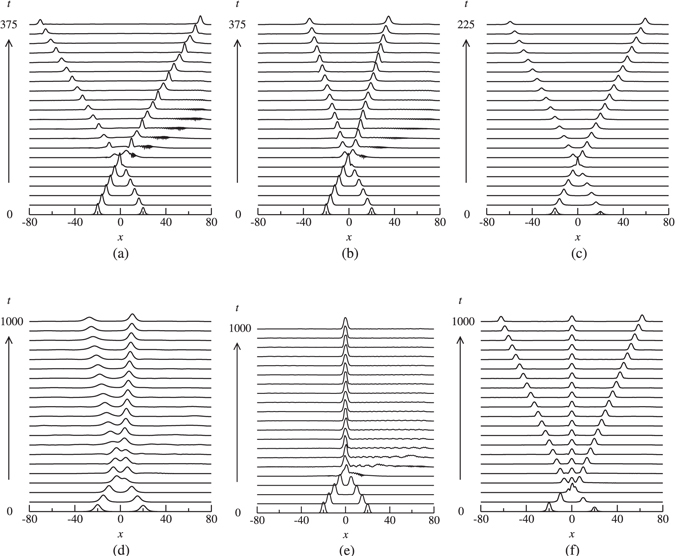
Examples of in-phase soliton collisions for *κ* = 10. (**a**) Symmetric separation with an increase in soliton velocity for *δ* = 0.2,  Ω= −9.93, *m* = 0.2 and *c* = 0.2; (**b**) symmetric separation with a decrease in soliton velocity for *δ* = 0.2, Ω = −9.93, *m* = 0.3 and *c* = 0.2; (**c**) symmetric separation with unchanged velocity for *δ* = 0.35, Ω = 10.31, *m* = 0.05 and *c* = 0.2; (**d**) Asymmetric separation for *δ* = 0.1, Ω = 10.31, *m* = 0.4 and *c* = 1; (**e**) merger into a zero velocity soliton for *δ* = 0.1, Ω = −10.10, *m* = 0.5 and *c* = 0.2; and (**f**) generation of three solitons with two moving ones and a quiescent soliton for *δ* = 0.2, Ω = 10.16, *m* = 0.5 and *c* = 0. Only |*u*| is shown.

**Figure 8:**
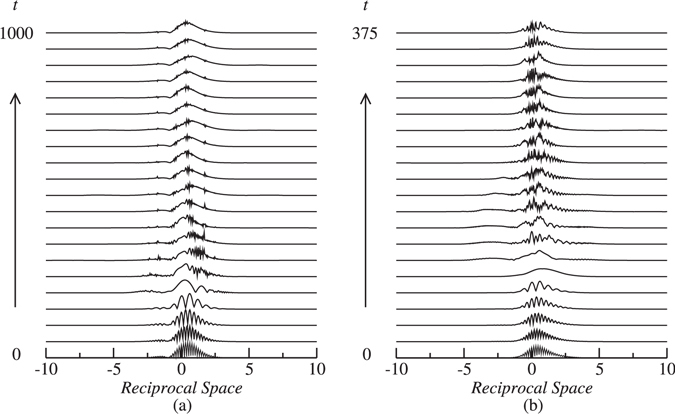
The amplitude spectrum of the collisions corresponding to (**a**) Fig. 7(e) and (**b**) Fig. 7(a). Only the spectrum for the *u*-component is shown.

The results of simulations have revealed that high velocity ($\delta \mathop{ > }\limits_{ \tilde {}}0.4$) in-phase (Δ*θ* = 0) collisions are generally quasi-elastic and result in the passage of solitons through each other without any conspicuous change in their velocities, whereas low velocity in-phase collisions are more inelastic and hence give rise to various outcomes. One possible outcome is emergence of two symmetrically separating solitons with increased, decreased or unchanged velocities. For example, the velocity of the emerging solitons has increased from 0.2 to 0.25 in Fig. 7(a), whereas it has decreased from 0.2 to 0.12 in Fig. 7(b). In both these cases, collisions are accompanied by a large amount of energy loss in the form of radiation. Figure 7(c) shows an example of a quasi-elastic collision in which soliton velocity remains unaffected as they collide. Another possible outcome is asymmetric separation of solitons, in which solitons undergo multiple collisions with subsequent splitting of solitons into two moving ones with unequal velocities (Fig. 7(d)). One of the most interesting outcomes is the formation of a quiescent soliton through collisions as shown in Fig. 7(e). As is shown in Fig. 7(f), collisions of solitons may also lead to the generation of three solitons, i.e. a quiescent one and two moving solitons propagating in opposite directions. A notable feature of such outcomes (i.e. 2 → 3 transformation) is that the amount of energy loss is considerably less than the other outcomes and the emerging solitons do not exhibit conspicuous breathing. In the case of *π*-out-of-phase collisions, solitons always bounce off each other with reversal in their direction of propagation. The spectra for Fig. 7(a,e) are shown in Fig. 8.

**Figure 9:**
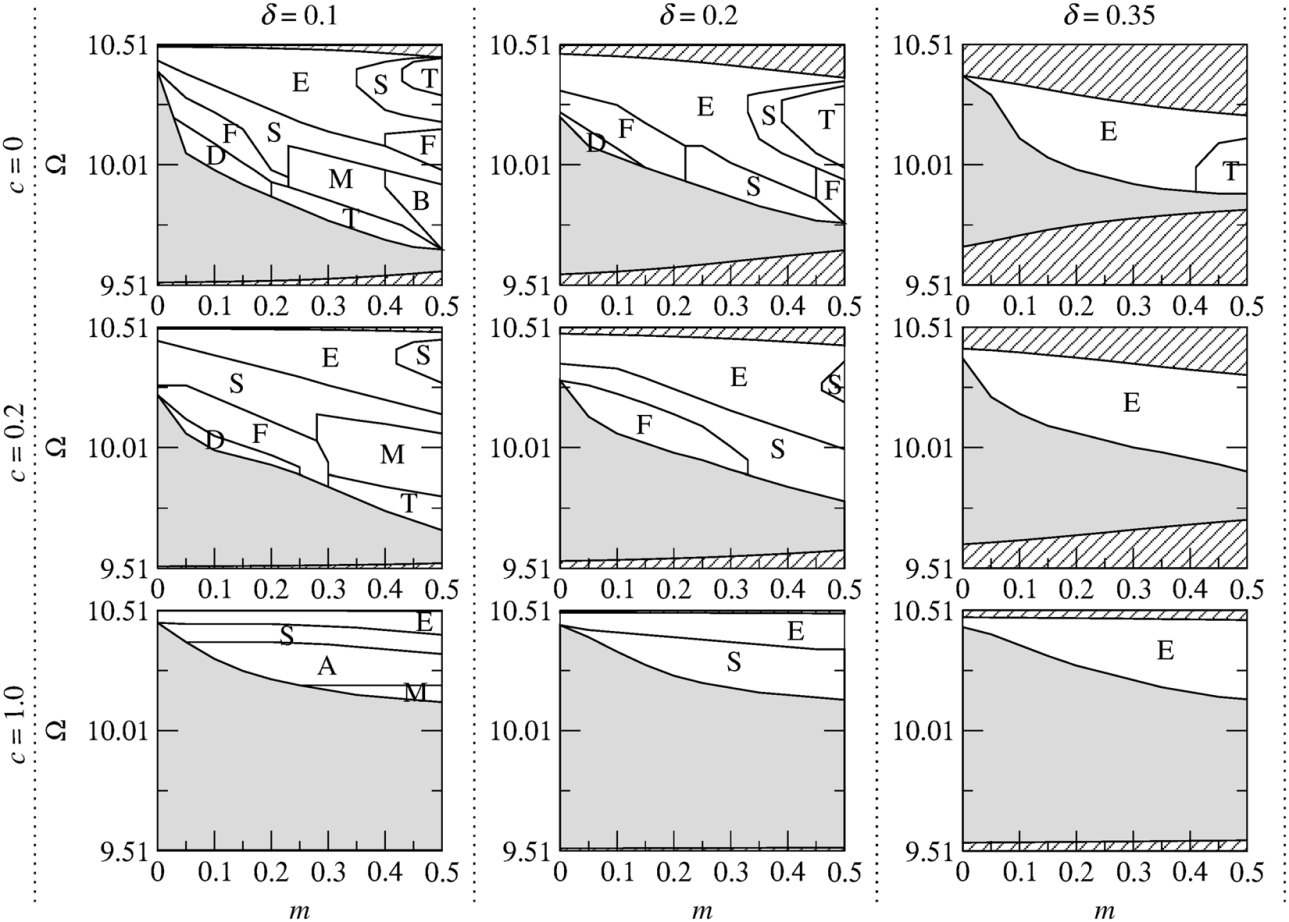
Collision outcome diagrams for in-phase solitons in the upper gap for several values of soliton velocity (increasing *δ* from left to right) and relative group velocity (increasing *c* from top to bottom) in the case of *κ* = 10. The regions are labeled as fast symmetric separation (F), slow symmetric separation (S), quasi-elastic separation (E), temporary bound state followed by separation (B), merger (M), three soliton generation (T) and destruction of soliton (D). The shaded areas indicate regions where solitons are unstable. The areas which lie outside the gap and consequently do not contain soliton solutions are shown by the diagonal lines.

**Figure 10:**
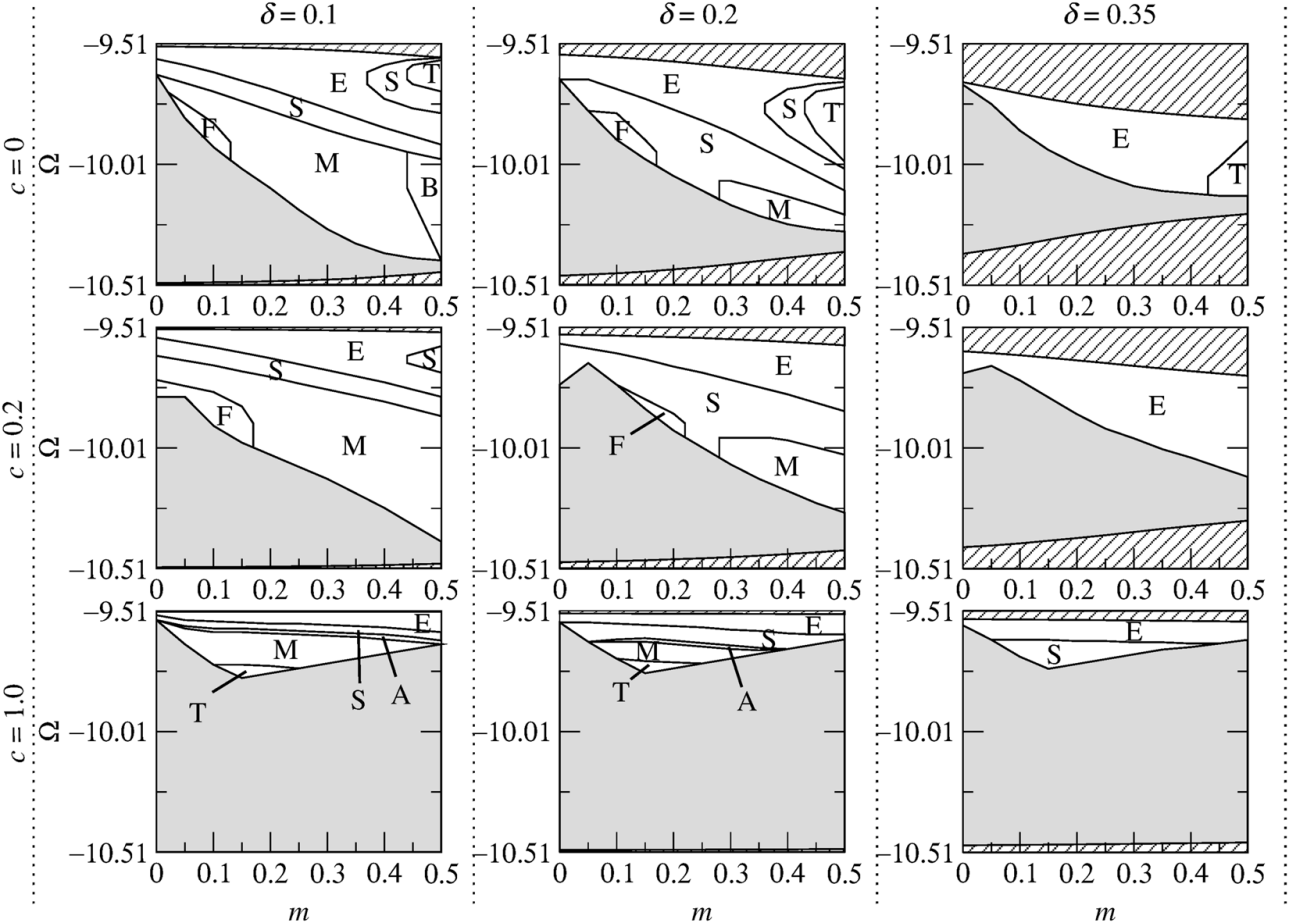
Collision outcome diagrams for in-phase solitons in the lower gap for several values of soliton velocity (increasing *δ* from left to right) and relative group velocity (increasing *c* from top to bottom) in the case of *κ* = 10. The regions are labeled as fast symmetric separation (F), slow symmetric separation (S), quasi-elastic separation (E), temporary bound state followed by separation (B), merger (M), three soliton generation (T) and destruction of solitons (D). The shaded areas indicate regions where solitons are unstable. The areas which lie outside the gap and consequently do not contain soliton solutions are shown by the diagonal lines.

In order to analyze the interplay of the parameters and their effect on the outcomes of the collisions, we have performed extensive systematic numerical simulations for different values of *κ*, *c* and *δ*. Through these simulations we have been able to identify the regions for various outcomes in the (*m*, Ω) plane. The results of the simulations for *κ* = 10 are summarized in Figs 9 and 10. As is shown in Figs 9 and 10, the richest collision dynamics is observed when *c* = 0 and *δ* = 0.1. In this case, a variety of outcomes may occur depending on the values of and *m*. This may, in part, be attributed to the fact that initially slow solitons have more “time” to interact compared with the faster ones. In both upper and lower gaps (Fig. 9), for a given *c*, increasing *δ* leads to the shrinkage and eventual disappearance of the merger region (region M). It is also worth noting that the region M is more pronounced in the lower gap than in its upper counterpart. In the upper gap, region T (i.e. 2 → 3 transformation) occurs for *c* = 0 and 0.2 primarily when dispersive reflectivity is strong. However, in the lower gap, the region T occurs for both moderate and strong dispersive reflectivity (see Fig. 10). In both the upper and lower gaps, the region E becomes more dominant as the velocity of the colliding solitons increases. As for collision-induced destruction of solitons (i.e. region D), it occurs only in the upper gap when *c* is small and $\delta \lesssim 0.22$.

**Figure 11:**
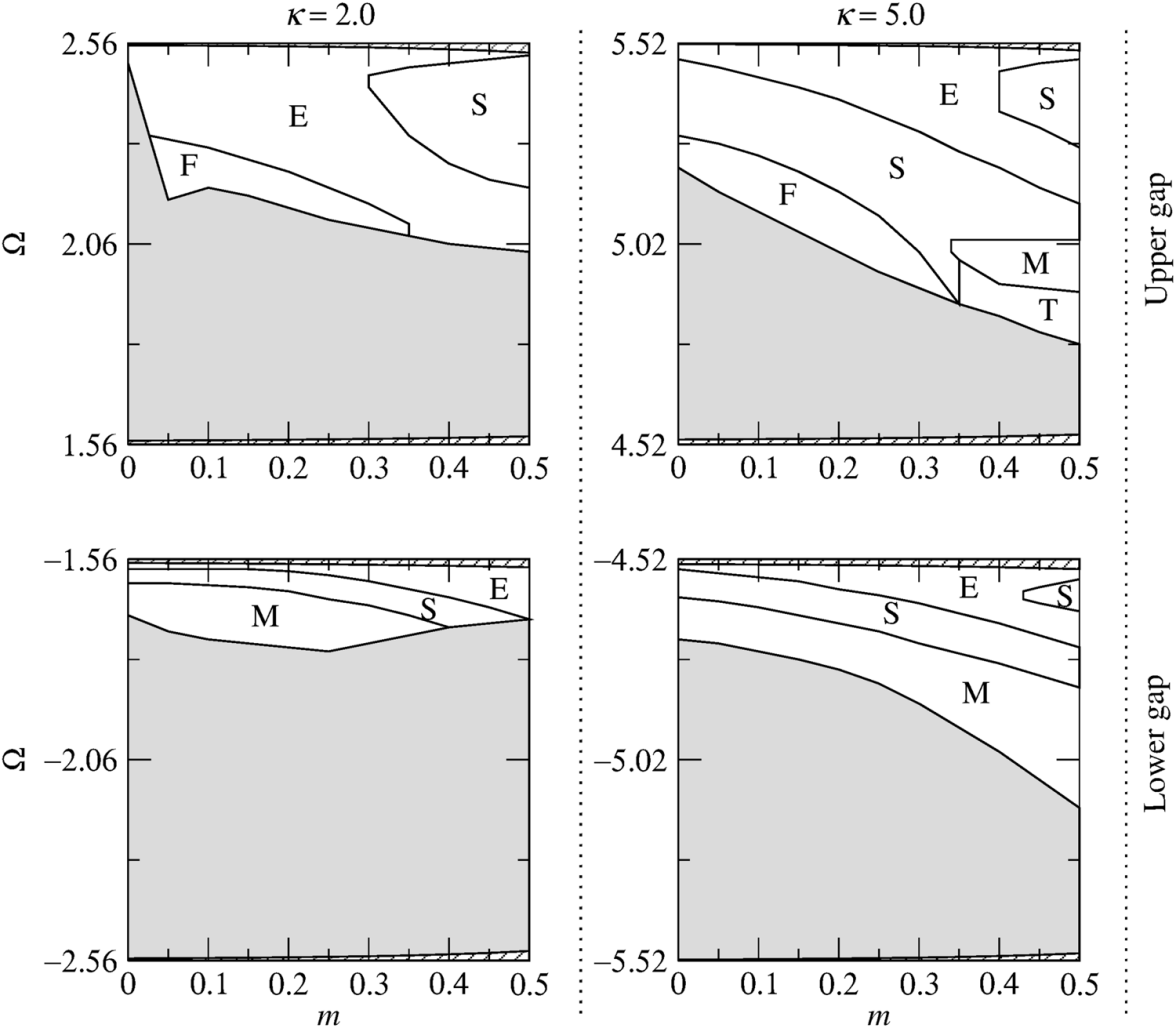
Collision outcome diagrams for in-phase solitons for different values of *κ* and *δ* = 0.1, *c* = 0.2. The regions are labeled as slow symmetric separation (S), fast symmetric separation (F), quasi-elastic separation (E), merger (M) and three soliton generation (T). The shaded areas indicate regions where solitons are unstable. The areas which lie outside the gaps and consequently do not contain soliton solutions are shown by the diagonal lines.

As was noted above, the collision dynamics are much richer for slow solitons. Therefore, in order to study the effect of *κ* on the outcomes of the collisions, we have set *δ* = 0.1 and *c* = 0.2 and simulated in-phase soliton-soliton collisions for different values of *κ*. The results of the simulations are displayed in Fig. 11. By comparing these results with those of *κ* = 10 (i.e., the diagrams corresponding to *δ* = 0.1 and *c* = 0.2 in Figs 9 and 10), some interesting characteristics can be extracted. One notable finding is that the merger (region M) and three soliton formation (region T) regions do not exist in the upper gap when *κ* = 2, however, these regions emerge as *κ* is increased from 2 to 5 and further expand at *κ* = 10. Also, in the lower gap, increasing *κ* from 5 to 10 leads to the emergence of the region F (i.e. fast symmetric separation).

**Figure 12:**
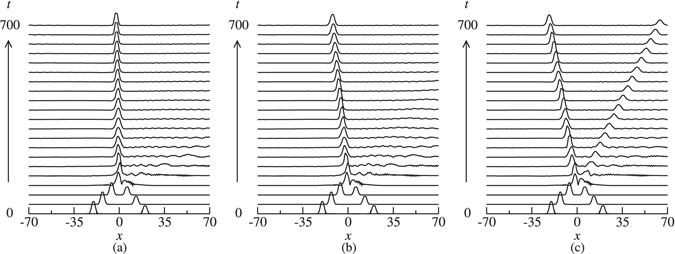
The effect of initial phase difference on the outcomes of collisions that lead to merger of solitons into a quiescent one as a result of in-phase collisions. The parameters are *κ* = 10, *δ* = 0.2, Ω = −10.15, *m* = 0.5, *c* = 0.2. (**a**) Δ*θ* = 1°, (**b**) Δ*θ* = 6°, and (**c**) Δ*θ* = 20°. Only |*u*| is shown.

In other models of BG solitons (e.g., the model of ref. 39) it has been shown that the collisions leading to merger of solitons are very sensitive to the initial phase difference. Our simulations demonstrate that in the present model this is not the case. More specifically, a small departure from Δ*θ* = 0 does not essentially prevent merger to occur, rather solitons merge into a very slow moving pulse as a result of collisions. Figure 12(a) demonstrates an example in which the collision with an initial phase difference Δ*θ* = 1° leads to fusion of solitons into a single soliton which travels at a very low velocity. The collision with an initial phase difference Δ*θ* = 6° in Fig. 12(b) still leads to merger, however, in this case, the generated pulse moves at a slightly higher velocity (~2% of the speed of light in the medium). A further increase in Δ*θ* (6° → 20°) in Fig. 12(c) gives rise to splitting of solitons into two moving ones with unequal velocities.

## Conclusions

In this paper, we have studied the existence, stability and collision dynamics of moving Bragg grating solitons in a semilinear dual core system where one core has the Kerr nonlinearity and a Bragg grating with dispersive reflectivity, and the other core is linear. It is found that when $\delta \ne 0$, the width of the upper/lower gap is dependent on *c*, *m* and *δ*. An empirical expression for the critical velocity at which the upper/lower gap closes has been determined. It is found that this critical velocity only depends on *c*. Moving soliton solutions exist as a continuous family of solutions in the upper and lower gaps. In order to determine the stability of the moving solitons, we have performed a systematic numerical stability analysis for various values of *κ*, *c*, and *δ* in the (*m*, Ω) plane. It is found that, for a given *κ* and *δ*, increasing *c* generally leads to the shrinkage of the stable region. Also, the area of the stable region in the (*m*, Ω) plane generally tends to reduce when soliton velocity *δ* is increased.

The dynamics and the outcomes of the collisions of counter-propagating solitons has been investigated through systematic numerical simulations. In the case of in-phase collisions, we have identified various outcome regions in the (*m*, Ω) plane for different values of *δ*, *c* and *κ*. High velocity in-phase collisions are generally quasi-elastic and result in the passage of solitons through each other without any conspicuous change in their velocities, whereas low velocity in-phase collisions can give rise to a number of interesting outcomes. One noteworthy outcome is the generation of a zero velocity soliton through merger of solitons or 2 → 3 transformation in certain parameter regions. An important finding is that the merger region in the (*m*, Ω) plane is more prominent in the lower gap than in its upper counterpart. Collisions of solitons can also lead to a decrease or an increase in their velocities as they pass through each other. In the case of *π*-out-of-phase collisions, solitons always bounce off each other.

### Data availability statement

The datasets generated during and/or analyzed during the current study are available from the corresponding author on reasonable request.
